# Continuous-flow self-supported seATRP using a sonicated microreactor†

**DOI:** 10.1039/d2sc03608h

**Published:** 2022-09-26

**Authors:** Suqi Zhang, Tanja Junkers, Simon Kuhn

**Affiliations:** Department of Chemical Engineering, KU Leuven Celestijnenlaan 200F 3001 Leuven Belgium simon.kuhn@kuleuven.be; Polymer Reaction Design Group, School of Chemistry, Monash University 19 Rainforest Walk, Building 23 Clayton VIC 3800 Australia

## Abstract

Continuous-flow simplified electrochemically mediated atom transfer radical polymerization (seATRP) was achieved for the first time without supporting electrolytes (self-supported) using a novel sonicated tubular microreactor. Polymerizations of different acrylic monomers were carried out under different applied currents. The reaction was fast with 75% conversion achieved at ambient temperature in less than 27 minutes. Results also showed good evolution of molecular weight and maintained narrow molecular weight distribution. The reaction rate can be further manipulated by tuning the applied current. Sonication under proper conditions was found to be able to significantly improve both reaction rate and controllability. Self-supported reactions also enable more environmentally friendly and cost-effective operations.

## Abstract 2

Continuous-flow self-supported seATRP was realized for the first time using a novel sonicated microreactor. This provides an alternative route to scale up the eATRP process and an opportunity for a more environmentally friendly synthesis.

## Graphical abstract

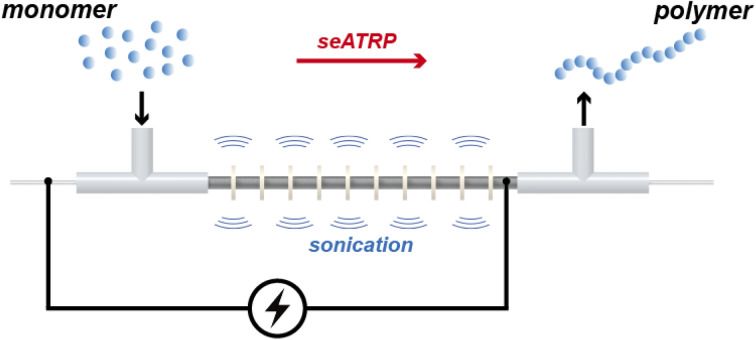

## Introduction

Continuous-flow reactions enable unique control over syntheses of both small molecules and macromolecules.^1,2^ Unlike traditional batch experiments, reagents are pumped through reactor channels with reactions taking place simultaneously. Many advantages of flow chemistry have been acknowledged, for instance, more convenient reaction condition control, straightforward scale-up strategy, higher reproducibility, and simplified handling of dangerous chemicals and/or intermediates.^3–8^ Specifically in electrochemistry, the typically very small electrode spacing in microreactor setups provides a more uniform current distribution, a better temperature control, a lower ohmic drop, and a very high surface-to-volume ratio.^9–12^ Additionally, this also provides the possibility of electrochemical synthesis without supporting electrolytes *i.e.*, self-supported synthesis, which can simplify downstream purification significantly.^13–15^

Among reversible deactivation radical polymerizations (RDRP), atom transfer radical polymerization (ATRP) is arguably among the most powerful ones.^16^ It has shown versatility in the synthesis of well-defined polymers with narrow molecular weight distribution (MWD), functionalities, and complex structures.^17^ Lately, electrochemically mediated atom transfer radical polymerization (eATRP) has attracted much interest due to its convenience in influencing polymerization behaviour externally.^18^ eATRP benefits from both the green nature of electrochemistry and the practical simplicity of ATRP. In an eATRP process, activators, normally a Cu(i)/ligand complex, are generated on the surface of the working electrode (WE) through direct electrochemical reduction of the stable deactivators (Cu(ii)/ligand complex). The activators then interact with the bulk solution and react with initiators, forming oxidized deactivators (Cu(ii)X/ligand) and a radical species. The radical species can then react with monomers and generate growing macroradicals. Subsequently, the propagating chains can react either with monomers or with Cu(ii)X/ligand to revert to their dormant form.^19^ Well-defined polymers are obtained by repeating the interplay and equilibrium between active and dormant chains as described above.

However, eATRP is still facing some limitations associated with the reactor setup. Conventionally, a three-electrode system is utilized to achieve eATRP. This means a reference electrode is required besides the working and counter electrodes. It is also vital to separate the counter electrode (CE) from the reaction mixture to avoid any anodic side reactions (divided cell). These limitations significantly increased the reactor complexity and hindered so far the translation of eATRP into flow. Efforts have been made by researchers to simplify the eATRP procedures, which led to the development of seATRP (s stands for simplified). In a typical seATRP reaction, a sacrificial anode (typically aluminium) is directly immersed into the reaction mixture, thus the polymerization can take place in an undivided cell and possibly by a galvanostatic process with only two electrodes involved. Another major hurdle for the application of eATRP is its limited scalability brought by the nature of electrochemistry, *i.e.*, the necessity of small reaction volumes between the electrodes.

Like most other electrochemical reactions, a rather large amount of supporting electrolyte needs to be added in seATRP to maintain a sufficient current and to lower the needed cell voltage.^18–20^ However, supporting electrolytes are generally not environmentally friendly and need to be removed during downstream purification. These drawbacks can be addressed when translating seATRP into flow utilizing microreactors.^14,21^ The straightforward scale-up strategy of microreactors could also provide an alternative solution to the scalability issue of eATRP.

### Design of the microreactor setup

A microreactor setup consisting of a tube and periodically spaced piezoelectric rings (piezorings) was developed by our group, and preliminary results demonstrate a promising application in electrochemistry.^22^ Meanwhile, the shear force formed near the reactor wall by acoustic streaming can also benefit the handling of highly viscous solutions which are rather common in polymer science and industry. We herein further developed this novel tubular microreactor setup, and seATRP was for the first time realized in continuous flow utilizing ultrasound. Results showed controlled monomodal molecular weight evolution throughout the reactions. Notably, the first realization of continuous flow seATRP enables a supporting-electrolyte-free process, which could bring both commercial and environmental benefits.

**Fig. 1:**
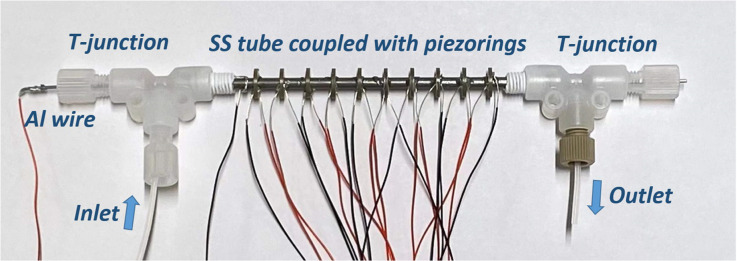
Photo of the microreactor.

As can be seen from Fig. 1, the microreactor comprises of a stainless-steel tube (od = 3 mm, id = 2.1 mm, *l* = 10 cm) and a straightened aluminium wire (od = 1 mm) fixed inside by junctions at both ends. Both sides of the tube were fixed to T-junctions. To maximize the sonicated volume of the setup the tightening nuts were shortened on the tube side (as can be seen in Fig. 1). The Al wire enters through the T-junction and is secured firmly by rubber septa and tightening nuts. 10 piezorings (id = 3 mm, od = 8 mm; 1 mm thick) were fixed to the tube periodically with a space of 5.5 mm. UV-cured glue was applied to keep the rings in place and to seal the very small air gap between the rings and the tube.

Fig. S1† schematically illustrates how these piezorings induce acoustic steaming. When an alternating voltage is applied to the flat faces of the piezoring, the ring will vibrate axially. *Via* Poisson's effect, these vibrations will also transmit radially through the capillary wall. At specific frequencies, the radial vibrations excite axial resonance modes in the liquid, and through viscous dissipation near the capillary wall a non-zero time-averaged Reynolds stress in the boundary layer is formed as a secondary effect. This Reynolds stress forces the liquid inside the boundary layer to drag the liquid outside the boundary layer into a circulatory motion.

## Results and discussion

In the first step the microreactor was analysed to identify the optimum sonication conditions (applied power and frequency). For this, the assembled microreactor setup was filled with acetonitrile and the resonance frequencies were identified using an impedance analyser. The first operable resonance frequency was found to be approximately 630 kHz. However, at this frequency the reaction solution (without initiator) turned cloudy after passing through the sonicated reactor even at a low applied power of 1 W. Gel permeation chromatography (GPC) results showed the formation of polymers with wide MWD (Fig. S2A†). Alternating acoustic pressure can cause the formation of cavitation bubbles. These bubbles initially oscillate at their resonance frequency and finally implode, which is a nearly adiabatic process. The estimated local temperature can reach up to 5000 K. Consequently, initiating free radicals can be generated *via* the direct degradation of acrylic monomers through a thermolytic process. Thus, we propose that this is the cause of the uncontrolled polymerization.^23,24^ Cavitation is less when higher frequencies are selected due to shorter rarefaction time.^23^ Therefore, a higher operating resonance frequency was selected and an applied power of 3 W at 1.35 MHz was found to limit radical formation while providing sufficient acoustic streaming, no polymer formation was found after reaction mixture passed through the sonicated microreactor without current applied.

Continuous flow seATRP was first carried out using the solution formula reported for conventional batch conditions ([M] : [I] : [CuBr_2_] :[ Me_6_TREN] = 150 : 1 : 0.09 : 0.09).^20^ However, the reaction stopped at very low conversion (∼10%) and the MWDs of products were wide (Fig. S2B†). After several attempts, we found excessive ligand necessary to realize the polymerization in a controlled manner. We hypothesize that this is due to the relatively high concentration of dissolved Al^3+^ since the volume of the solution electrolyzed is very small in a microreactor systems. While in batch experiments, this influence is not decisive due to its much larger volume.^25^ The dissolved Al^3+^ might then compete with the Cu catalyst for the ligands and thus result in a loss of active catalyst, leading to uncontrolled polymerization. Thus, sufficient excessive ligand was added to rule out the effect of ligand loss. The amount of excess ligand added for each experiment was calculated according to the theoretical amount of dissolved Al^3+^ ions corresponding to the longest residence time to be operated at (see Section 2.4 in ESI†).

**Fig. 2:**
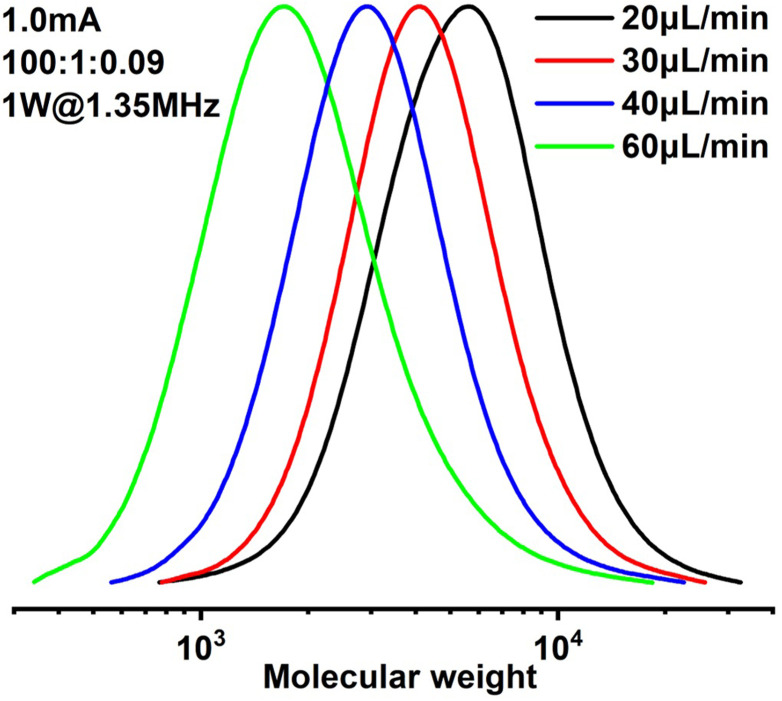
Molecular weight evolution of polymerization of methyl acrylate.

**Fig. 3:**
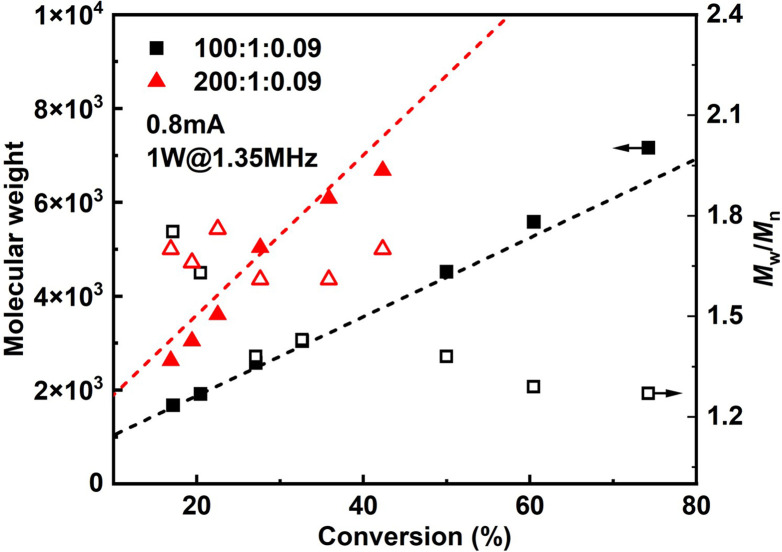
*M*_*n*_ and *M*_w_/*M*_*n*_*versus* conversion of MA polymerizations by seATRP.

Since it is very difficult to integrate a reference electrode in a microreactor system, a series of methyl acrylate (MA) polymerizations were carried out under galvanostatic condition, and ethyl α-bromoisobutyrate (EBiB) was used as initiator. As the potential of the working electrode cannot be precisely controlled, an amount of Cu catalyst loss is inevitable. Therefore, higher catalyst loadings were applied to improve the controllability of the reaction. Total reflection X-ray fluorescence (TXRF) was also carried out to quantify the residual amount of Cu catalyst (Table S2†). As can be seen in Fig. 2, MWs evolved well in all cases and MWDs were relatively narrow. The MW grows linearly with the conversion and is close to the theoretical value (Fig. 3). We noticed that the MWDs of the obtained polymer products were generally higher than those obtained from batch experiments. This follows the general kinetics as residence time distribution (RTD) in continuous flow reactors is normally wider than that in conventional batch reactors due to the parabolic velocity profile.^21^ Polymerization of butyl acrylate (BA) was also performed (Fig. S4†). However, due to the limitation of the current setup (small reactor volume and single step galvanostatic process), quantitative conversion was difficult to reach.

Results of polymerizations of two different acrylic monomers are summarized in Table 1. Higher catalyst loadings can further narrow the MWD entry 1 and 3. It is also observed that a higher catalyst is required to control the polymerization of BA entry 10 and 11. And when dealing with more viscous solutions entry 9, 1 W of applied sonication power is found insufficient, thus the power was increased to 3 W. The operational window of sonication is found to be wider in this case, due to more viscous dissipation. It is also worth noting that all the polymerizations were carried out at ambient temperature.

**Table 1:** Sonicated continuous flow self-supported seATRP of different monomers at ambient temperature (∼23 °C) in the developed reactor

Entry	Monomer	[M] : [I] :[ Cat.]	*I*_app_ (mA)	Flowrate (μL min^−1^)	Residence time (min)	Conv. (%)	*M*_*n*,GPC_ b (Da)	*M*_*n*,theo_ (Da)	*Đ*
1	MA	100 : 1 : 0.09	0.8	20	13.4	38	3600	3466	1.36
2	MA	100 : 1 : 0.09	0.8	10	26.8	75	6800	6652	1.38
3	MA	100 : 1 : 0.12	0.8	20	13.4	57	4900	5102	1.30
4	MA	100 : 1 : 0.12	0.8	15	17.9	65	5400	5600	1.24
5	MA	200 : 1 : 0.09	0.8	15	17.9	50	8200	8804	1.55
6	MA	100 : 1 : 0.09	1.2	40	6.7	21	1800	2003	1.58
7	MA	100 : 1 : 0.09	1.0	40	6.7	26	2300	2433	1.48
8	MA	100 : 1 : 0.09	0.8	40	6.7	27	2200	2519	1.41
9^a^	MA	500 : 1 : 0.20	0.8	10	26.8	56	20 700	24 300	2.04
10	BA	100 : 11 : 0.12	0.8	15	17.9	64	8700	8398	1.57
11	BA	100 : 1 : 0.15	0.8	20	13.4	59	8000	7707	1.37

a Applied sonication condition: 1 W@1.35 MHz except 3 W@1.35 MHz.

b Measured by GPC using PMMA (entry 1–8, 10, 11) or PS (entry 9) calibration standards.

**Fig. 4:**
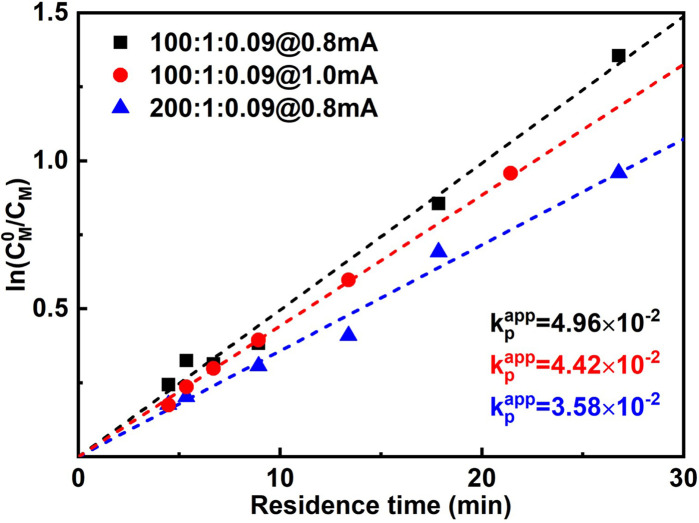
First-order kinetic plot of different polymerization conditions.

When the current applied (*I*_app_) is increased from 0.8 mA to 1.0 mA, the conversion is instead slightly lower, while *Đ* is significantly larger. This was more obvious when 1.2 mA was applied. It can also be seen from the first-order kinetic plot (Fig. 4) that the apparent propagation rate constant (*k*^app^_p_) decreases slightly from 4.96 × 10^−2^ to 4.42 × 10^−2^ when *I*_app_ increases from 0.8 mA to 1.0 mA ([M] : [I][CuBr_2_] = 100 : 1 : 0.09). We assume this is due to that more negative WE potential (to maintain higher current) also favours side reactions, which could lead to lower faradaic efficiency. Not surprisingly, higher *I*_app_ lead to less controlled reactions (higher dispersities), as there are more activated chains present at the same time. We also noticed that too high *I*_app_ also leads to gas generation. Void segments can be observed at the outlet FEP tubing. This could also be attributed to the possible electrolysis of reactant(s). The plot of conversion as a function of residence time is depicted in Fig. S5.†

**Fig. 5:**
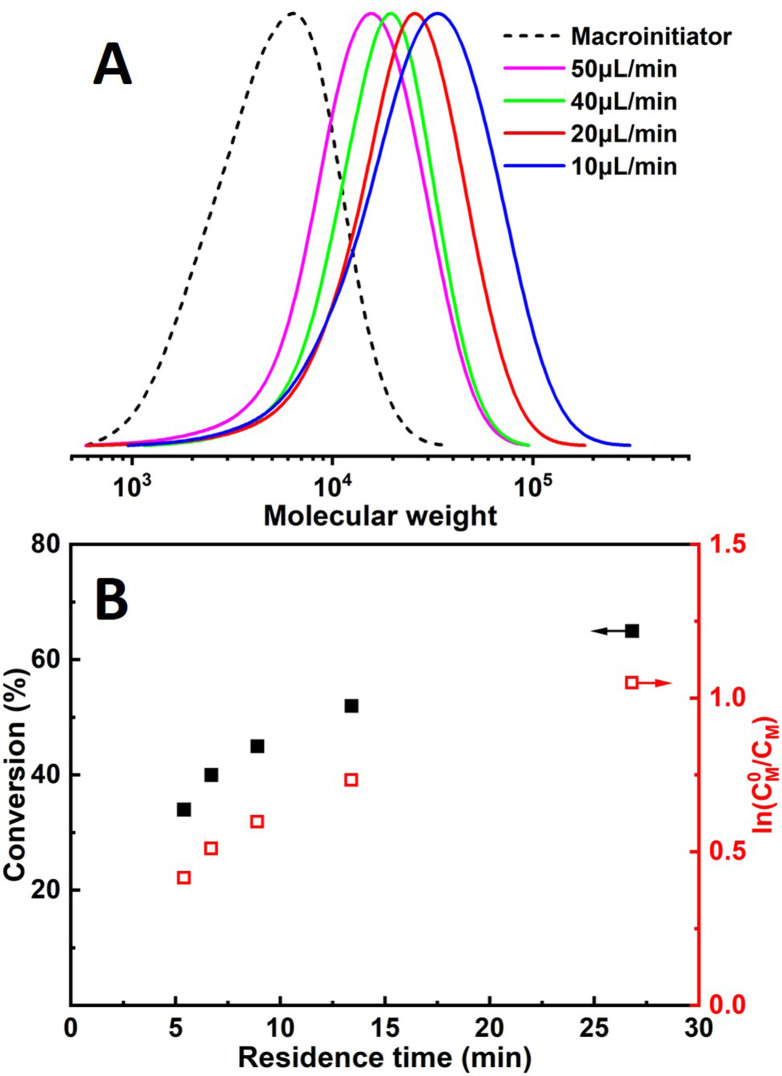
(A)MWD evolution and (B)first-order kinetic plot of chain-extension experiments.

Chain extension was performed to evaluate the chain-end fidelity and overall quality of control. PMA macroinitiator (*M*_*n*_ = 3900, *Đ* = 1.56) was synthesized by continuous flow seATRP using the developed microreactor setup, followed by extension with the same monomer (MA) for the sake of avoiding GPC calibration issues. Linear first-order kinetics were still observed (Fig. 5B) and a relatively clean peak shift can also be seen from GPC traces (Fig. 5A).

A sample with high monomer conversion (*M*_*n*_ = 2500, 83% monomer conversion) was also prepared and further analyzed by electrospray ionization mass spectrometry (ESI-MS) to obtain more detailed information on the end group fidelity (Fig. S8†). From the zoom-in spectrum of repeating unit with 1920–1995 *m*/*z*, it can be seen that the most abundant species are sodium and potassium adducts of the expected polymer product. Yet, a small fraction of proton-terminated polymer can also be observed (C and D), this might be an artifact stemming from ionization. However, this may also indicate a small amount of chain transfer product. Peaks specific for bimolecular termination were largely absent (note that the difference between a proton-terminated species and a product from termination is that termination requires two products to form when occurring *via* disproportionation). Notably, unidentified peaks were found in the vicinity of the main product peaks (E). The isotope pattern of the peaks indicates they contain bromine, meaning they are still living chains, and are likely the product from initiation of a so far unknown species (probably electrochemically initiated). This alternative initiation could also explain why dispersity is slightly increasing at longer reactor residence times. All peak assignments can be found in Table. S1.†

**Fig. 6:**
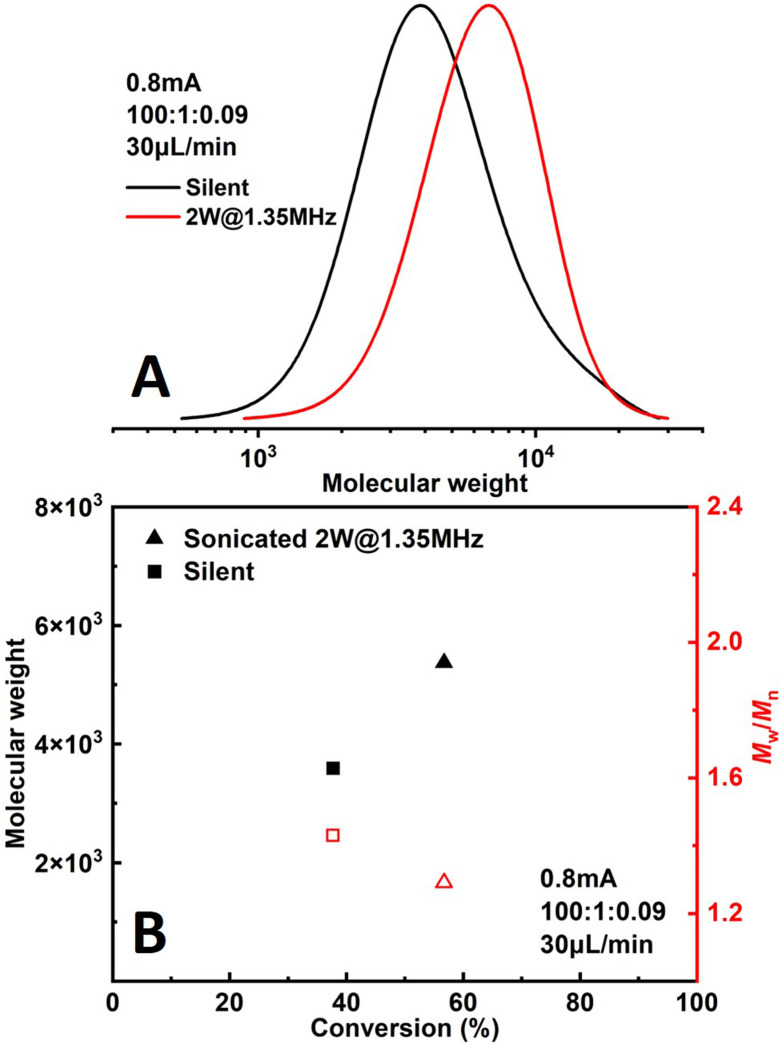
Comparison of polymerizations of methyl acrylate under silent and sonicated conditions.

**Fig. 7:**
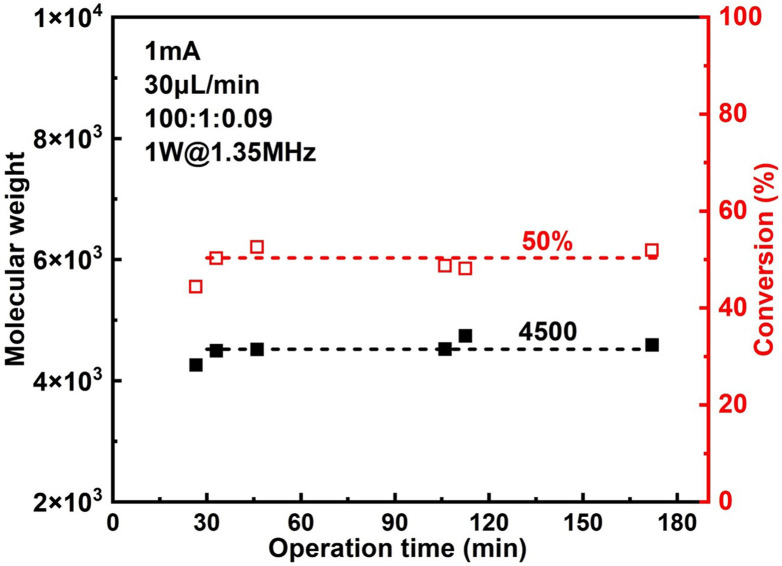
Stability of the novel microreactor setup.

The effect of sonication is shown more directly in Fig. 6. When the flowrate is set to 30 μL min^−1^ (corresponding to a residence of ∼8.9 min), conversion increased significantly from 38% to 57% while *Đ* dropped from 1.43 to 1.27 under 2 W of sonication at 1.35 MHz (Fig. 7B). A much narrower MWD can be seen from GPC results in Fig. 7A, notably, the formation of polymers with extremely large MW (less controlled part) is to a large extent diminished. This can be attributed to more efficient deactivation of propagating chains thanks to the better mass transfer induced by acoustic streaming. The stability of the developed microreactor setup was also examined by conducting the reaction for approximately 3 h with *I*_app_ = 1.0 mA at 30 μL min^−1^ flowrate. The conversion and corresponding MW are found to be lower in the first 45 min, indicating a lower current efficiency. After this period, the reaction reaches a basically steady state Fig. 7. Since we are using non-noble metal (SS304) as the WE, there would inevitably be a thin film of oxides on the surface of the electrode.^26^ We assume that during the first 45 min a fraction of the current is consumed by the reduction of these surface oxides, which causes a lower current efficiency.

**Fig. 8:**
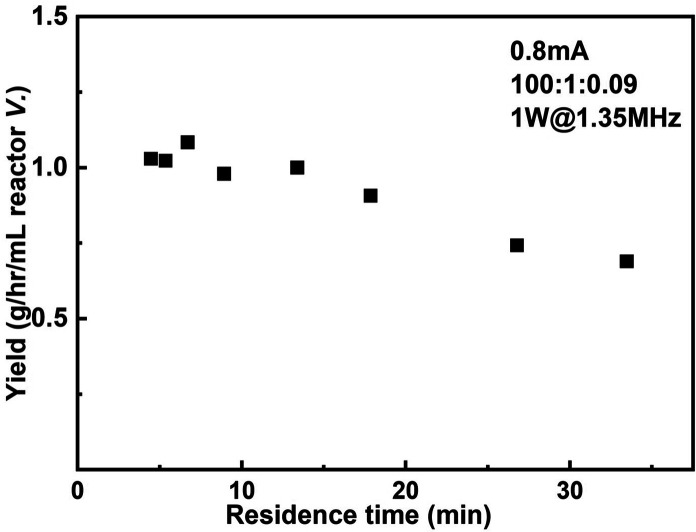
Productivity of the reactor setup as a function of residence time.

The productivity of the reactor setup as a function of residence time when 0.8 mA current and 1 W sonication power were applied is shown in Fig. 8. The largest productivity 1.08 g h^−1^ mL^−1^ reactor volume is obtained when operated at 40 μL min^−1^ flowrate, corresponding to a residence time of 6.7 min. In the case of highest conversion (75%, residence time = 26.8 min), the productivity still maintains 0.74 g h^−1^ mL^−1^ reactor volume.

## Conclusions

In summary, seATRP was for the first time achieved in flow with the help of a novel sonicated tubular microreactor. While the space time yield of the current reactor is still relatively low, this work paves the way for overcoming the scaling issue of eATRP.

The unique merits of the sonicated microreactor and continuous operation enabled a self-supported reaction, bringing both economic and environmental benefits. Results showed polymerizations of both MA and BA were well-controlled, with MW evolutions close to the theoretical value, and successful chain extension reactions. A high endgroup fidelity of the living chain end is also demonstrated. Furthermore, the reaction was fast even at ambient temperature (∼23 °C), namely ∼80% conversion in less than 27 min (entry 2, Table 1), providing the possibility of more energy-efficient synthesis. It was also found crucial to add excess ligand, due to the competition for ligand between the copper catalyst and dissolved aluminium ions released from the sacrificial anode during the electrolysis, as well as some possible destabilizing side reactions that could cause catalytic activity loss. These limitations would be an interesting aspect to improve in future research to increase the environmental benefits of this setup. A higher catalyst load compared to batch experiments was also needed to obtain a narrower MWD. Meanwhile the dispersity of the product is generally higher when compared with conventional eATRP processes reported in literature due to the intrinsic properties of continuous-flow reactor (Table 1). But the reaction rate is significantly higher even at ambient temperature. The introduction of acoustic streaming into continuous flow polymerization is also of great interest when handling viscous solutions, which are fairly common in polymer sciences. As it is intrinsic that the WE potential cannot be precisely controlled in galvanostatic processes, the setup can be further developed and optimized by expansion to multistep where different currents can be applied to improve the electrochemical reduction selectivity. This proposed setup could offer inspiration for continuous precise polymer synthesis in both academia and industry.

## Author contributions

S. Z. contributed to the full extent of this work, ranging from the experimental setups, data acquisition, interpretation, analysis, to the writing of the manuscript. T. J. was involved in the discussions, some analyses as well as writing and reviewing the original draft. S. K. was involved in the discussions, writing, and reviewing of the original draft, and is responsible for funding acquisition and project administration.

## Conflicts of interest

There are no conflicts to declare.

## Supplementary Material

SC-013-D2SC03608H-s001
