# Supplementary material for: Continuous-flow self-supported seATRP using a sonicated microreactor
Source: Chem Sci. 2022 Sep 26;13(42):12326–31. doi: 10.1039/d2sc03608h (PMC9628926; doi:10.1039/d2sc03608h)
Supplement: SC-013-D2SC03608H-s001 [file SC-013-D2SC03608H-s001.pdf]

Electronic Supplementary Material (ESI) for Chemical Science.
This journal is © The Royal Society of Chemistry 2022

Electronic Supplementary Information

Continuous-Flow Self-Supported seATRP Using a Sonicated Microreactor

Suqi Zhang[1], Tanja Junkers[2] and Simon Kuhn[1]

[1]Department of Chemical Engineering, KU Leuven,

Celestijnenlaan 200F, 3001 Leuven, Belgium

[2]Polymer Reaction Design Group, School of Chemistry, Monash University,

19 Rainforest Walk, Building 23, Clayton, VIC 3800, Australia

Table of Contents

1 Materials and Reactor setup

2 Experimental Procedures

2.1 General Polymerization Procedures

2.2 Sonication Conditions

2.3 Rinse Procedure

2.4 Dissolved Al^{3+} Calculation

2.5 Chain Extension Experiments

3 Measurements and Analysis

4 Results and Discussion

4.1 Molecular Weight Evolutions

4.2 Conversion as a Function of Residence Time

4.3 Chronopotentiometry

4.4 ^{1}H NMR Spectra of Polymers Synthesized

4.5 ESI-MS Data

4.6 Total Reflection X-ray Fluorescence Analysis for Residual Cu Amount

5 References

1 Materials and Reactor Setup

Methyl acrylate (MA, >99%), butyl acrylate (BA, >99%), acetonitrile, copper (II) bromide ($CuBr_2$, 99%), and ethyl α-bromoisobutyrate (EBiB, 98%) were purchased from Merck. Tris[2-(dimethylamino)ethyl]amine (Me_6TREN, 98%, TCI) was purchased from VWR.

Al wire (1mm) and stainless-steel tube (od=3mm, id=2.1mm) were purchased from Goodfellow. T-junctions (P633 Tee Assembly, ETFE) were purchased from IDEX Health and Science. Piezorings (SMR837T1411) were purchased from STEMINC. UV glue was purchased from Revell. The tube and rod were both connected electrically to a potentiostat (Stanford Research Systems EC301) via wire soldered on. Coupled piezorings were connected to an E&I 2100 L amplifier, which amplifies the sinusoidal signal generated by a Rigol DG1032 wave generator.

How the piezorings induce acoustic streaming is schematically shown below:

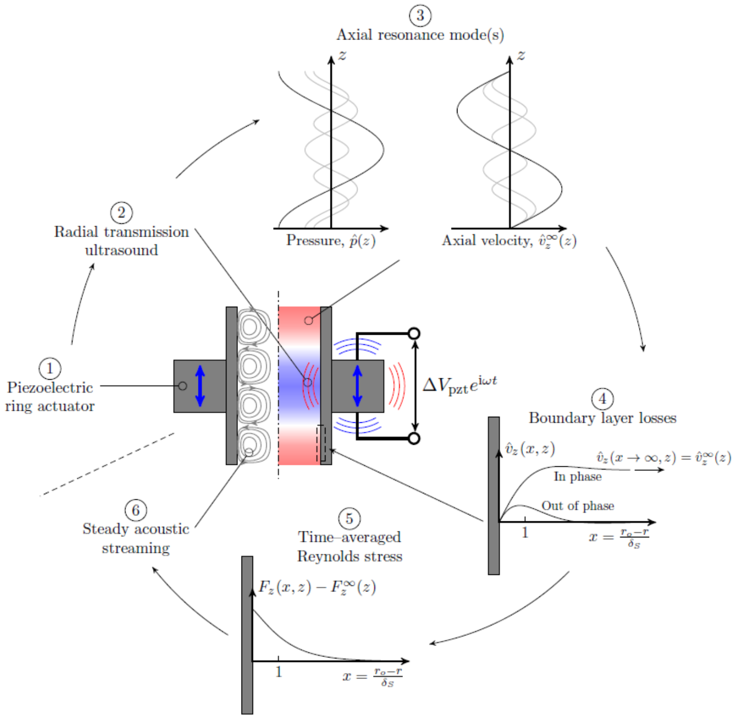

Figure S1. Schematic representation how (1) an axially poled piezoelectric actuator vibrates and (2) *via* Poisson's effect also transmits radial vibrations through the capillary wall. These radial vibrations excite (3) axial resonance modes in the liquid filled channel, which (4) are attenuated in a thin Stokes boundary layer. The attenuation (5) induces a Reynolds stress in this boundary layer, which (6) drags the liquid outside the boundary layer into a steady streaming motion[1].

2 Experimental Procedures

2.1 General Polymerization Procedures

3mL of acetonitrile, 3mL of MA, 48.6μL of EBiB, 26.6μL of Me_6TREN and 0.398mL of $CuBr_2/Me_6TREN$ stock solution (0.1M in acetonitrile) were added in a Schlenk flask, the dissolved oxygen was removed by 5 freeze-pump-thaw cycles. The degassed solution was then transferred to syringe and pumped into the reactor setup using a syringe pump (Harvard Apparatus PHD Ultra). Formula of polymerizations with different monomer selection or [M]:[I]:[Cat.] ratios were adjusted correspondingly. Samples were collected after four residence time and conversions were measured using gravimetric analysis. The molecular weights (Mn and Mw) and molecular weight distribution (Mw/Mn) were determined by gel permeation chromatography (GPC) measurements (with PS standard curve).

2.2 Sonication Conditions

Resonance frequencies of solution filled reactor setup were measured using an impedance analyzer (SinePhase 16777k). For each experiment, signal frequency and amplitude were set on the signal generator (1.350MHz, 150mVpp for 1W cases and 210mVpp for 2W cases) and amplified by an E&I 2100 L amplifier. A ventilator was utilized to remove the heat generated by the piezorings.

2.3 Rinse Procedure

After each sample was collected, a special rinsing procedure was performed to obtain reproducible results. The procedure includes the following steps: i) 10mL 1M HNO_3 was pumped through the reactor at 1mL/min under 5W sonication at 635kHz, then the pump was stopped, and the nitric acid solution was kept in the reactor for 5min. After this ii) flush with 10 mL Milli-Q water at 1mL/min iii) and finally with another 5mL acetonitrile also at 1mL/in.

2.4 Dissolved Al^{3+} Calculation

The molar concentration of dissolved Al^{3+} can be calculated using the equation below:

$$C_{Al} = \frac{I \cdot t}{3F \cdot V}$$

where I is the current applied, t stands for the residence time, V stands for the volume of the reactor, and F is the Faraday constant. As in this research the reactor volume stays unchanged, the equation can be also written as:

$$C_{Al} = \frac{I}{3F \cdot Q}$$

where Q denotes the applied flow rate.

For instance, when 0.8mA current was applied and the smallest flow rate of 10μL/min (0.1667μL/s) was chosen, the molar concentration can be calculated to be:

$$C_{Al} = \frac{I}{3F \cdot Q} = \frac{0.8 \cdot 10^{-3}\ (A)}{3 \cdot 96485.33\ \left(\frac{C}{mol}\right) \cdot 0.1667 \cdot 10^{-6}\ \left(\frac{L}{s}\right)} = 16.58\ (mmol/L)$$

Hence, a minimum of 16.58mmol/L of excess ligand will be added to compensate for the aluminum dissolution.

2.5 Chain Extension Experiments

A low DP PMA macroinitiator (Mn=3900, *Đ*=1.56) was prepared by seATRP using the novel microreactor. Reaction conditions: [MA]:[EBiB]:[Cat./L]=100:1:0.12, 0.8mA current was applied and the flowrate was set to be 30μL/min, corresponding to a residence time of 8.9min. 1W sonication power at 1.35MHz was applied. The effluent from the reactor was collected and then passed through a column filled with basic alumina to remove copper catalyst. After that the solution was left in a fume hood for 48h to recover the macroinitiator, and the macroinitiator was further dried under vacuum for 4hr.

The chain extension was carried out under the following procedures and conditions: 539.75mg recovered macroinitiator, 4mL MA, 0.405mL 0.109M catalyst stock solution and 50μL of Me_6TREN were added into 4mL MeCN. The dissolved oxygen was removed by 5 freeze-pump-thaw cycles. Then the degassed solution was transferred to a syringe and pumped into the reactor setup using the same procedures in general polymerizations mentioned above. As the solution viscosity is higher in chain extension experiments due to larger polymer MWs, higher power sonication was applied, namely 3W at 1.35MHz.

Samples were collected after four residence time and conversions were measured using gravimetric analysis. The molecular weights (Mn and Mw) and molecular weight distribution (Mw/Mn) were determined by gel permeation chromatography (GPC) measurements (with PS standard curve).

3 Measurements and Analysis

Molecular weights and distributions were measured by GPC (SHIMADZU LC20) equipped with a differential refractive index detector (RID) and with THF eluent at 35°C, 1.0mL/min flowrate. Agilent PLgel 3μm MIXED-E 300×7.5mm column was used and the column were calibrated using 7 linear poly(methyl methacrylate) (PMMA) standards (Mn=580-27 810). For higher molecular weight case (Table1, Entry 8), Agilent PLgel 5μm MIXED-C 300×7.5mm column was used with calibration of 12 linear polystyrene (PS) standards (Mn=1 230-195 600). Mark-Houwink-Kuhn-Sakurada constants of relevant polymers were applied to correct measured molecular weights (PS: K=0.0141, α=0.7; PMA: K=0.0102, α=0.74; PBA: K=0.0122, α=0.7).

^{1}H NMR (400 MHz) spectra were measured on a Bruker Advance III HD 400 spectrometer using $CDCl_3$ as solvent.

Chronopotentiometry was done using the Stanford Research Systems EC301 potentiostat mentioned above.

Electrospray ionization mass spectrometry (ESI-MS) was performed using an LCQ Fleet mass spectrometer (ThermoFischer Scientific) equipped with an atmospheric pressure ionization source set in nebulizer assisted electrospray mode. Calibration was performed in the mass/charge (m/z) range of 220–2000 with a standard solution containing caffeine, L-methionyl-arginyol-phenylalanylalanine acetate H_2O and Ultramark 1621. A constant spray voltage of 5 kV was employed with an applied nitrogen dimensionless auxiliary gas flowrate of 3 and a dimensionless sheath gas flowrate of 3. The capillary voltage, tube lens offset voltage and capillary temperature were set to 25, 120 V, and 275 °C, respectively. A 250 μL aliquot of polymer solution with concentration of 10 $\mu g \cdot mL^{-1}$ was injected with HPLC grade THF and methanol (THF:methanol 3:2) as solvent.

4 Results and Discussion

4.1 Molecular Weight Evolutions

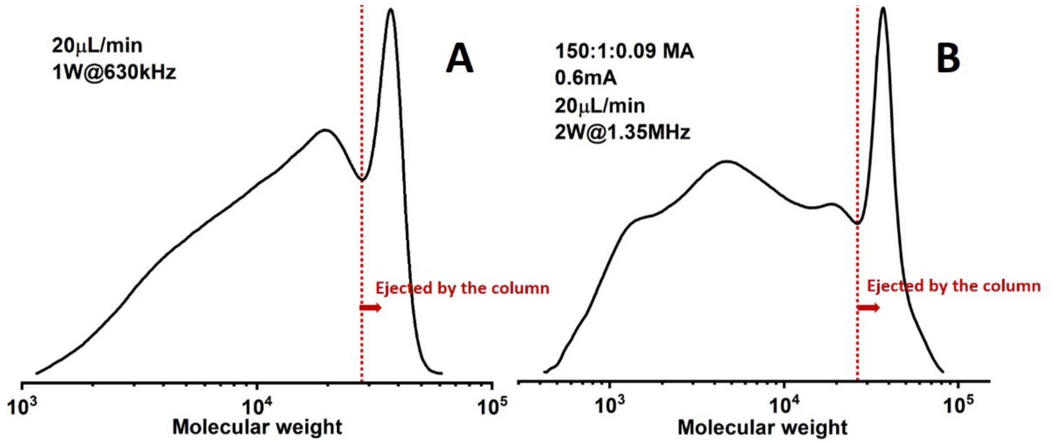

Figure S2. Polymerization was found to be uncontrollable when (A)630kHz sonication was applied or (B)excess ligand was not added.

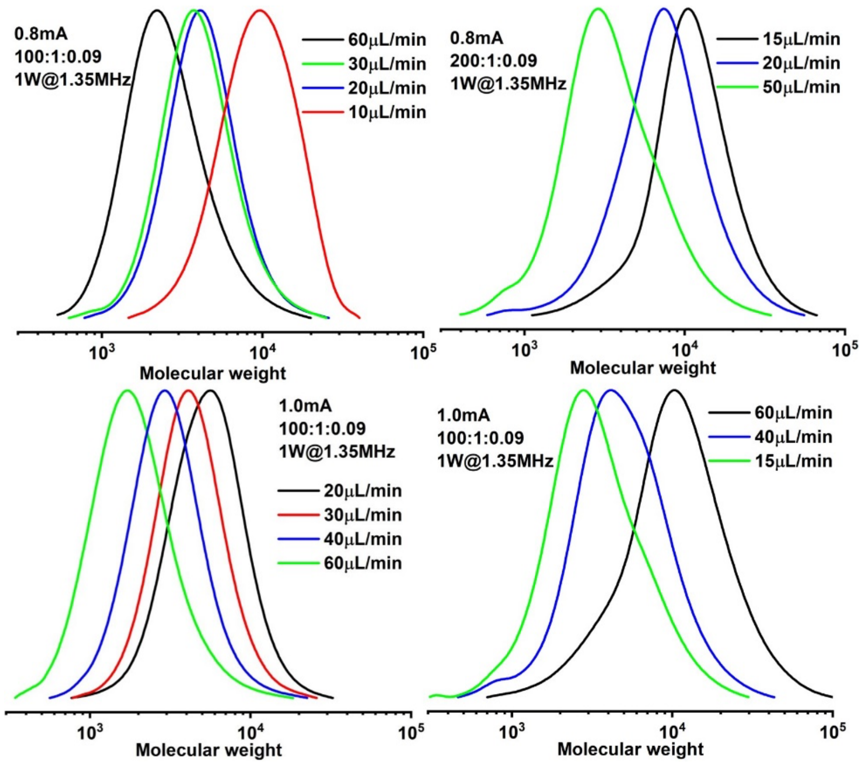

Figure S3. Molecular weight evolutions of polymerization of methyl acrylate.

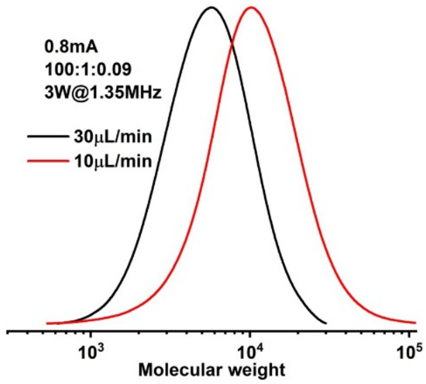

Figure S4. Molecular evolution of polymerization of butyl acrylate.

4.2 Conversion as a Function of Residence Time

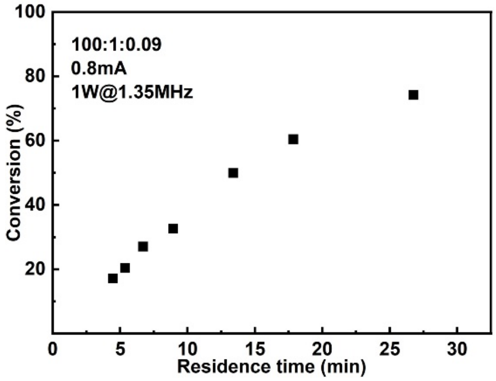

Figure S5. Conversion as a function of residence time.

4.3 Chronopotentiometry

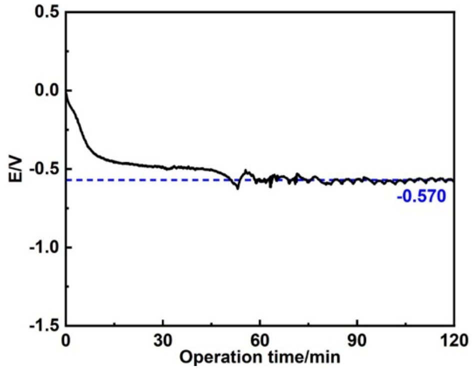

Figure S6. Chronopotentiometry of galvanostatic polymerization of methyl acrylate. I_{app}=0.8mA, [M]:[I]:[Cat.]=100:1:0.09.

4.4 ^{1}H NMR Spectra of Polymers Synthesized

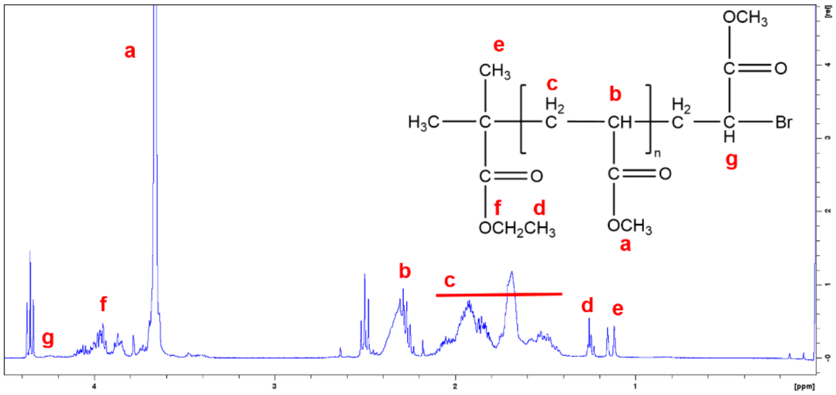

Figure S7. 400MHz ^{1}H NMR spectrum of purified PMA by continuous-flow self-supported seATRP (in $CDCl_3$).

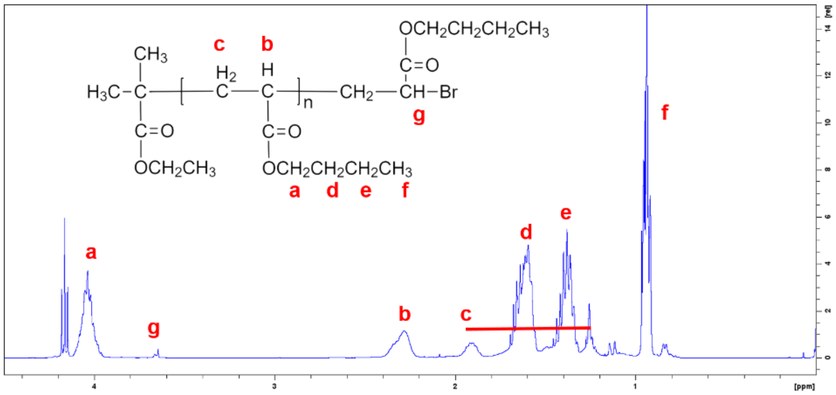

Figure S8. 400MHz ^{1}H NMR spectrum of purified PBA by continuous-flow self-supported seATRP (in $CDCl_3$).

4.5 ESI-MS Data

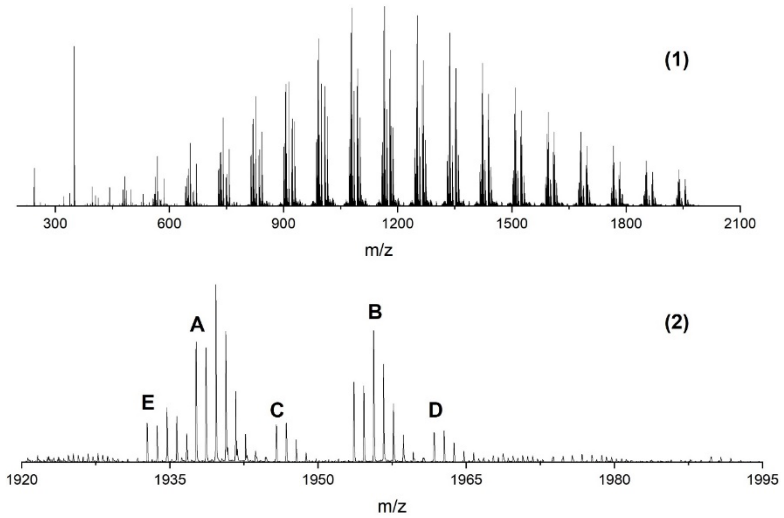

Figure S9. (1) Full ESI-MS spectrum and (2) zoom-in of one repeating unit of purified PMA by continuous-flow self-supported seATRP.

Table S1. ESI-MS peak assignments

Peak	α end group	ω end group	Units MA	Ion	m/z, exp	m/z, theo	δ/Da
A	EBiB	Br	20	Na^+	1937.67	1937.72	0.05
B	EBiB	Br	20	K^+	1953.54	1953.69	0.15
C	EBiB	H	20	Na^+	1945.78	1945.84	0.06
D	EBiB	H	20	K^+	1961.77	1961.81	0.04
E	Unknown	Br?	20	Unknown	1932.71	-	-

4.6 Total Reflection X-ray Fluorescence Analysis for Residual Cu Amount

Table S2. Residual Cu amount after reaction, measured by TXRF. Original solution: [M]:[I]:[Cat.]=100:1:0.09, monomer: MA. Cu concentration in original solution is measured to be 240mg/L. Sonication: 1W@1.35MHz.

**Sample**	**$I_{app.}$ (mA)**	**Flowrate (µL/min)**	**Residual Cu concentration (mg/L)**	**Residual Cu (%)**
1	0.8	10	160	67
2	0.8	20	204	85
3	0.8	40	220	92
4	1.0	40	188	78
5	1.2	40	170	71

5 References

1 S. Fransen, *Analysis of Laminar Flow Electrochemical Reactors: Self-supported Synthesis and the Effects of Ultrasound*, PhD thesis, KU Leuven, Leuven, Belgium, 2019.
